# The role of interdependent self‐construal in mitigating the effect of conspiratorial beliefs on vaccine acceptance

**DOI:** 10.1111/bjso.12836

**Published:** 2024-12-09

**Authors:** Yingli Deng, Cynthia S. Wang, Gloria Danqiao Cheng, Jennifer A. Whitson, Benjamin J. Dow, Angela Y. Lee

**Affiliations:** ^1^ Durham University Business School Durham University Durham UK; ^2^ Kellogg School of Management Northwestern University Evanston Illinois USA; ^3^ Anderson School of Management University of California Los Angeles California USA; ^4^ Cox School of Business Southern Methodist University Dallas Texas USA

**Keywords:** conspiratorial beliefs, COVID‐19, interdependent self‐construal, prosocial motivation, vaccine acceptance, vaccine hesitancy

## Abstract

Infectious diseases pose significant challenges to public health, leading to illness and even death. Vaccinations are vital for protecting society, yet beliefs in conspiracy theories related to infectious diseases increase vaccine hesitancy. This paper delves into vaccination decisions in the context of COVID‐19, which continues to strain the health care system. While past research focuses on countering conspiratorial beliefs with cognitive persuasion interventions, we propose a social intervention as an alternative. Our novel intervention seeks to mitigate the effects of conspiratorial beliefs by fostering individuals' interdependent self‐construal – viewing oneself in the context of social relationships. Interdependent self‐construal was operationalized in multiple ways (measured in Studies 1, 2 and 3; manipulated to test causality in Studies 4 and 5). Conspiratorial beliefs were also manipulated in Study 5. The results show that the association between conspiratorial beliefs and vaccine hesitancy is weakened among individuals whose interdependent self‐construal is more accessible. Moreover, this effect was mediated by prosocial motivation. We discuss the implications of our findings for developing and communicating health policies and propose potential contexts where this intervention may be relevant, thereby providing valuable insights into enhancing societal well‐being in the face of conspiratorial beliefs.

## INTRODUCTION

The threat of infectious diseases to public health is a pressing concern, especially as climate change increases the likelihood of viruses jumping from wildlife and livestock to human populations and causing pandemics (Carlson et al., 2022; de Oliveira & Tegally, 2023). COVID‐19 infected over 775 million people and caused more than 7 million fatalities worldwide (COVID‐19 Cases | WHO COVID‐19 Dashboard, n.d.). Vaccinations can reduce outbreaks of infectious diseases such as COVID‐19 (Andrews et al., 2022; Lopez Bernal et al., 2021; Townsend et al., 2023), combat new variants (Krammer & Ellebedy, 2023) and alleviate the strain on the health care system (Hamson et al., 2023).

However, vaccine effectiveness is highly dependent on people's willingness to receive them (Böhm & Betsch, 2022; Dawson & Verweij, 2007). Unfortunately, vaccine hesitancy persists (Frankovic, 2021; Kleitman et al., 2023; Limbu & Huhmann, 2023), with one dominant cause grounded in conspiracy theories related to infectious diseases (Jolley & Douglas, 2014; Shapiro et al., 2016). Indeed, individuals who hold COVID‐19 conspiratorial beliefs have been reported to be less confident in the efficacy of COVID‐19 vaccines (Howard & Davis, 2023; Romer & Jamieson, 2020) and are less likely to receive the vaccine (Akther & Nur, 2022; Shakeel et al., 2022).

**FIGURE 1:**
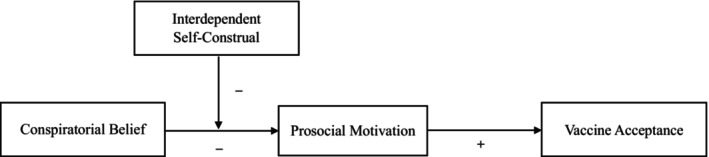
Theoretical model.

This paper introduces a novel factor that may minimize the effects of these beliefs. We propose that the association between conspiratorial beliefs and vaccine acceptance can be weakened by making salient people's interdependent self‐construal, which involves seeing oneself through the lens of one's social relationships. Moreover, we propose that this occurs because conspiratorial beliefs suppress prosocial motivation – the desire to safeguard and promote the welfare of others (Grant & Berg, 2011) – which in turn is positively associated with vaccine acceptance (Enea et al., 2023). Thus, by reframing how people think about themselves in terms of their social ties, a salient interdependent self‐construal should weaken the negative relationship between conspiratorial beliefs and prosocial motivation, resulting in increased vaccine acceptance. The full theoretical model is depicted in Figure 1.

Our paper seeks to contribute to research on conspiratorial beliefs by highlighting a social intervention that may blunt their effects. Past interventions have focused on cognitive persuasion efforts, such as providing counterarguments to conspiracy theories. Those that have shown some success are limited by the need to reach individuals before being exposed to conspiracy theories – a challenge when such theories are already widespread (for a review, see Dow et al., 2021). Our framework contributes by showing that the negative impact of conspiratorial beliefs on vaccine acceptance is transmitted through reduced prosocial motivation and that this impact can be weakened when one's interdependent self‐construal becomes more accessible – an intervention that does not require engaging directly with conspiratorial beliefs. That is, changing how individuals view themselves can effectively mute the adverse effects of conspiratorial beliefs on their motivational desire to help others, thereby increasing vaccine acceptance.

This research contributes more widely to our understanding of how beliefs and attitudes influence health‐protective behaviours and enhance societal welfare. Many vaccination campaigns seek to effect behavioural change by focusing on individual benefits. For example, to reduce vaccine hesitancy, some campaigns highlight the science of vaccine development and safety measures (Hing et al., 2022), while others emphasize how vaccines minimize health threats to the individual (Motta et al., 2021). In contrast, by making individuals' interdependent self‐construal salient, our proposed intervention aims to effect behaviour change by refocusing individuals' self‐concept around their relational connections. With successful pandemic management dependent on a sufficiently large number of citizens adhering to public health recommendations (Chu et al., 2020), our proposed approach adds to the growing work on improving coordination in collective response efforts to crises (Kafadar et al., 2023).

### Theoretical development

#### Conspiratorial beliefs and vaccine acceptance

Individuals hold conspiratorial beliefs ‘when they accept explanations that attribute causality to multiple actors secretly working together to achieve harmful, or even malevolent, goals’ (Whitson et al., 2019, p. 4). Conspiratorial beliefs have a broad array of adverse effects on society across different domains, including reduced political engagement (Butler et al., 1995), non‐adherence to health‐protective advice (e.g. not using condoms; Grebe & Nattrass, 2012) and decreased health‐seeking intentions (Natoli & Marques, 2021). These beliefs also heighten prejudice (Kofta & Sedek, 2005), science denial (Goertzel, 2010) and even extremism (van Prooijen et al., 2015).

People are drawn to conspiratorial beliefs because they offer a potential path to satisfying psychological needs. For example, people may endorse conspiratorial beliefs in an attempt to make sense of a chaotic world, with the hope of restoring a sense of control over their lives or reducing uncertainty (Douglas et al., 2017, 2019; Dow et al., 2023; Whitson et al., 2022). Individuals also adopt conspiratorial beliefs to satisfy social motives, such as the desire to maintain positive views of themselves and their groups (Douglas et al., 2017, 2019). For example, conspiratorial beliefs allow group members to compare themselves favourably to outgroups and provide external targets to blame for their own group's low status (van Prooijen, 2024).

The motivation to reduce uncertainty is heightened during societal crises like COVID‐19. The effects of COVID‐19 persist years after the World Health Organization declared it a pandemic, with new variants of the virus posing a continual public health threat (McVernon & Liberman, 2023; Meyerowitz et al., 2024). The pandemic gave rise to misinformation and conspiracy theories (Dow et al., 2021, 2023) – such as those suggesting the virus was developed as a bioweapon or it was simply a hoax exaggerated for political reasons – which have spread almost as quickly as the disease itself (Clark et al., 2008; Fotakis & Simou, 2023; Imhoff & Lamberty, 2020). For example, one poll revealed that a quarter of Americans surveyed believed powerful people likely planned the outbreak (Schaeffer, 2020), and nearly 25% of Americans surveyed believed that the virus was engineered in a laboratory, as did over 70% of respondents in Greece, 46% in Turkey and 36% in the United Kingdom (Tsamakis et al., 2022).

Research has established that individuals who endorse health care–related conspiracy theories are less likely to engage in recommended health‐protective behaviours (Douglas et al., 2017; Dunn et al., 2017; Jolley & Douglas, 2014). Endorsers of COVID‐19 conspiratorial beliefs are no exception, with research showing that such beliefs are tied to vaccine hesitancy and non‐compliance with masking, social distancing and vaccination recommendations (Allington et al., 2021; Bertin et al., 2020; Romer & Jamieson, 2020; Stasielowicz, 2022).

We suggest that one reason COVID‐19 conspiratorial beliefs are negatively associated with vaccine acceptance is that they decrease prosocial motivation – the desire to safeguard or invest effort for the well‐being of others (Grant & Berg, 2011). More specifically, conspiratorial beliefs direct individuals' attention to potential threats and malevolent forces in their environment, which heightens their sense of fear and anxiety. This, in turn, leads individuals to prioritize self‐preservation over helping others (Hornsey et al., 2021; Pummerer et al., 2023). Indeed, it has been shown that individuals who hold conspiratorial beliefs tend to score higher in trait measures such as Machiavellianism, which is characterized by a strategic self‐interested focus (March & Springer, 2019), and narcissism (Cichocka et al., 2016), which is associated with selfishness and low concern for others (Krizan & Herlache, 2018). Both general conspiratorial beliefs (e.g. beliefs that information is being hidden from us) and specific COVID‐19 conspiratorial beliefs have been shown to reduce the desire to help others or make personal sacrifices to prevent the spread of COVID‐19 (Enea et al., 2023; Moon & Travaglino, 2021). Furthermore, individuals prone to conspiratorial beliefs were more likely to indicate that their motives for adhering to the COVID‐19 lockdown measures were to protect themselves, rather than others (Marinthe et al., 2020).

We suggest that people's prosocial motivation has important implications for vaccine acceptance. Prosocially motivated individuals exhibit a stronger commitment to prosocial behaviour (Ilies et al., 2006) and are more outcome oriented, actively seeking ways to benefit others (Grant, 2007; Grant & Mayer, 2009). Indeed, heightened prosocial motivation prompts greater efforts to safeguard the welfare of others (Schwartz et al., 2000). Research shows that prosocial motivation predicts vaccination intentions (Böhm et al., 2016; Böhm & Betsch, 2022; Enea et al., 2023; Li et al., 2016). Participants primed with a prosocial (vs. pro‐self) motivation were more likely to self‐isolate and vaccinate against COVID‐19 (Böhm & Betsch, 2022; Enea et al., 2023; Heffner et al., 2021; Hornsey et al., 2021; Jung & Albarracín, 2021; Oleksy et al., 2022). We propose that COVID‐19 conspiratorial beliefs are negatively associated with vaccine acceptance by suppressing prosocial motivation. More formally stated:

Hypothesis 1 Conspiratorial beliefs are negatively associated with vaccine acceptance.

Hypothesis 2 Prosocial motivation mediates the relationship between conspiratorial beliefs and vaccine acceptance.

### The moderating role of interdependent self‐construal

Given the severity of public health consequences, it is important to identify interventions to counter the effect of conspiratorial beliefs on vaccine acceptance. Most interventions identified in previous research adopt a cognitive approach designed to inoculate individuals against misinformation before they are exposed to a conspiracy theory. For example, ‘pre‐bunking’ involves presenting individuals with arguments debunking a conspiracy theory before they are exposed to arguments in favour of it (Jolley & Douglas, 2017). Other approaches involve inducing critical thinking immediately before exposure to a conspiracy theory (Bago et al., 2022; Bertolotti & Catellani, 2023) or illustrating how to recognize manipulative, persuasive techniques used to spread conspiracy theories (Roozenbeek et al., 2022). One prerequisite for these approaches is reaching the intended audience before exposure to conspiracy theories. Unfortunately, given how widespread COVID‐19 conspiratorial beliefs already are (Schaeffer, 2020), the effectiveness of these preventative methods is necessarily limited. The alternative of altering people's existing COVID‐19 conspiratorial beliefs is remarkably challenging (Bago et al., 2022; Dow et al., 2021; Jolley & Douglas, 2017) – research shows that most cognitive interventions designed to debunk anti‐vaccine conspiracy theories by providing scientific evidence have limited effectiveness (Helfers & Ebersbach, 2023; O'Mahony et al., 2023). Some emerging evidence suggests that fact‐based persuasion is possible, but requires those endorsing conspiratorial beliefs to engage in in‐depth, personalized conversations. This may be challenging to accomplish among populations who are suspicious of outside attempts to engage with their beliefs (Costello et al., 2024). As a result, people exposed to conspiracy theories often remain unvaccinated (Ecker et al., 2022; Helfers & Ebersbach, 2023; Jolley & Douglas, 2017). Importantly, none of these approaches address the crucial link between conspiratorial beliefs and reduced prosocial motivation.

In light of the limitations of cognitive approaches to counter the impact of conspiratorial beliefs, Dow et al. (2021) call for exploring interventions prioritizing social factors. There is suggestive evidence that this route is promising. For example, leveraging social norms to alter conspiratorial beliefs (e.g. presenting actual, as opposed to perceived, rates of peer endorsement of vaccine‐related conspiracy theories; Cookson et al., 2021) has been shown to increase vaccination intentions. In this research, we focus on the effects of increasing individuals' connections to others. We propose that one approach to mitigate the negative impact of COVID‐19 conspiratorial beliefs on vaccine acceptance is to enhance the accessibility of one's interdependent self‐construal.

The concept of self‐construal originates from research examining cultural differences in how one perceives the self (Markus & Kitayama, 1991). Broadly speaking, some individuals view themselves as autonomous beings (i.e. they have an independent self‐construal), while others view themselves as part of a larger social collective (i.e. they have an interdependent self‐construal). In essence, an individual's self‐construal is an ‘interpretive framework for understanding the social world’ that moulds their cognitions and motivations (Gardner et al., 1999, p. 321). People with a more accessible interdependent self‐construal see themselves through the lens of their relationships with others; they tend to derive positive feelings from developing and maintaining close relationships (Cross et al., 2000). In contrast, those with a more accessible independent self‐construal focus on their individuality and tend to develop positive feelings from highlighting their unique selves and characteristics (Cross et al., 2000).

We propose that an interdependent self‐construal can help mitigate the negative relationship between conspiratorial beliefs and vaccine acceptance. Consistent with Hypothesis 1, the belief that COVID‐19 was intentionally created and spread is negatively related to vaccine acceptance among individuals whose interdependent self‐construal is less accessible. However, individuals whose interdependent self‐construal is more accessible, who tend to view themselves within the context of a group, are more likely to consider the potential impact of their decisions on others despite their conspiratorial beliefs. In this case, they may prioritize reducing the potential harm caused by resisting vaccination and the subsequent risk of infecting others with COVID‐19, even if they still have concerns about the origins of the virus. Indeed, research shows that individuals whose interdependent self‐construal is more accessible consider vaccinations a more critical measure for protecting the collective good (Geiger et al., 2022; Li et al., 2016). As such, an accessible interdependent self‐construal would moderate the relationship between conspiratorial beliefs and vaccine acceptance.

Hypothesis 3 Interdependent self‐construal weakens the negative relationship between conspiratorial beliefs and vaccine acceptance.

We further posit that interdependent self‐construal moderates the effect of conspiratorial beliefs on vaccine acceptance by weakening their negative impact on the individual's prosocial motivation to protect the welfare of others. As discussed earlier, conspiratorial beliefs prompt individuals to perceive threats in their environment and focus on self‐preservation (Pummerer et al., 2023). When individuals' interdependent self‐construal is less accessible, the negative effects of conspiratorial beliefs on prosocial motivation persist because they are more self‐focused and often downplay the implications of their actions (or inactions) on others. In contrast, individuals whose interdependent self‐construal is more accessible are mindful of their relationships with others and, hence, are more inclined to fulfil their social responsibilities (Lee et al., 2000). Even though conspiratorial beliefs may continue to highlight threats in the environment for these individuals, their orientation towards group concerns buffers the detrimental effects of these beliefs on prosocial motivation. Thus, we propose:

Hypothesis 4 Interdependent self‐construal weakens the negative relationship between conspiratorial beliefs and prosocial motivation.

To the extent that reduced prosocial motivation is associated with reduced vaccine acceptance, the effect of conspiratorial beliefs on reduced vaccine acceptance via reduced prosocial motivation is likely weakened for those whose interdependent self‐construal is more accessible. Thus, we propose that making salient one's interdependent self‐construal will dampen the negative relationship between conspiratorial beliefs and vaccine acceptance by weakening the effects of conspiratorial beliefs on prosocial motivation:

Hypothesis 5 Interdependent self‐construal moderates the indirect relationship between conspiratorial beliefs and vaccine acceptance via prosocial motivation.

### Overview of research

We tested our hypotheses across five studies. Study 1 examined H1 and H3 using a measure of vaccine acceptance and self‐reported vaccination behaviour as the dependent variables. Study 2 tested the full model using updated vaccine (i.e. ‘booster’) acceptance as the dependent variable. Study 3 examined data across three cultural contexts to test H1 and H3. Study 4 manipulated the moderator to further examine causality and tested H1 and H3. Finally, Study 5 manipulated the independent variable and the moderator to test the full model through a causal paradigm involving a fictitious disease.

All study materials are in Appendix S1. The analysis code and data for variables described in the paper are available online: https://osf.io/9baxk/?view_only=b6382f98de4d4d07af2571d46eec4972. The studies received Institutional Review Board approval, and informed consent was obtained before we collected data. We conducted post‐data‐collection sensitivity analyses using G*Power for the first four studies to determine whether our sample size provided sufficient power to test our hypotheses (Faul et al., 2007), assuming two‐tailed tests and an alpha of .05. For Studies 1 and 4, the model included three predictors: interdependent self‐construal, conspiratorial beliefs and the interaction term between these two variables. For Study 2, the model included four predictors: interdependent self‐construal, conspiratorial beliefs, prosocial motivation and the interaction term between conspiratorial beliefs and interdependent self‐construal. For Study 3, the model included five predictors: culture, interdependent self‐construal, conspiratorial beliefs, the interaction term between conspiratorial beliefs and culture and the interaction term between conspiratorial beliefs and interdependent self‐construal. The results revealed that our sample sizes were sufficiently powered at 80% to detect the minimum effect sizes of *f* = .20 for Study 1 (*N* = 290), *f* = .14 for Study 2 (*N* = 643), *f* = .16 for Study 3 (*N* = 487) and *f* = .12 for Study 4 (*N* = 702). For Study 5, which was pre‐registered (https://aspredicted.org/21D_SZ7), we ran an a priori power analysis using the effect size from Study 4 (*f* = .12), which also included a manipulation, in G*Power with the ‘ANOVA: Fixed effects’ function. The result suggested a sample size of *N* = 547 (actual *N* = 557 after exclusions) for a two‐tailed test (*α* = .05) with 80% power.

## STUDY 1

Study 1 examined our prediction that conspiratorial beliefs were negatively related to vaccine acceptance (H1) and that this relationship would be weaker for individuals whose interdependent self‐construal is more accessible (H3).

### Participants and procedure

We recruited participants using Prolific in October 2021 (when vaccines were widely available). Participants were 303 adults residing in the United States who were compensated $1.50 for completing the study. We excluded 10 participants who indicated that they did not have access to vaccines and 3 who did not disclose their vaccination status. Our final sample was 290 participants (gender: 40.3% women, 58.6% men, 1.1% non‐binary individuals; *M*_*age*_ = 32.81 years, *SD*_*age*_ = 8.58; education: 7.6% had some high school education or a high school degree, 8.6% had an associate degree, 13.1% had some college education, 36.9% had an undergraduate degree, 33.8% had a graduate degree; race: 66.6% Caucasian, 23.8% Black, 4.8% Hispanic, 2.4% Asian, 2.4% other).

### Measures

#### Conspiratorial beliefs

Participants responded to a five‐item measure of conspiratorial beliefs regarding COVID‐19 adapted from Imhoff and Lamberty (2020) (e.g. ‘COVID‐19 was purposefully created in, and released from, a biochemistry lab’, 1 = *strongly disagree* to 5 = *strongly agree*; *α* = .89).^1^

#### Interdependent self‐construal

Participants responded to a five‐item measure of interdependent self‐construal developed by Singelis (1994) (e.g. ‘I will sacrifice my self‐interest for the benefit of the group I am in’, 1 = *strongly disagree* to 7 = *strongly agree*; *α* = .76).

#### Vaccine acceptance

Three items were adapted from Di Martino et al. (2020) to measure vaccine acceptance (e.g. ‘During the pandemic, it has been very important to me to get a vaccine’, 1 = *strongly disagree* to 7 = *strongly agree*; *α* = .95).^2^

#### Vaccination status

Participants reported their vaccination status: Those not vaccinated were coded as 1, those who were partially vaccinated were coded as 2 and those who were fully vaccinated were coded as 3.^3^

### Control variables

We measured participants' age, gender, race, political affiliation and education level, as these variables may affect their attitudes towards vaccination. Additionally, to demonstrate the effects of interdependent self‐construal as distinct from the potential effects of independent self‐construal, we assessed participants' independent self‐construal using the established five‐item measure from Singelis (1994; e.g. ‘I do my own thing, regardless of what others think’, *α* = .75). The pattern of results remained the same with or without these control variables. We report results without controls below and results with controls in Appendix S1.

### Results

Table 1 shows Study 1 means, standard deviations and correlations. We used Hayes's (2017) SPSS PROCESS macro Model 1 with 5000 bootstrapped samples and a 95% confidence interval to test our hypotheses.^4^ In our regression analyses, we mean‐centred conspiratorial beliefs and interdependent self‐construal. The results are reported below and in Table 2.

**TABLE 1:** Means, standard deviations and correlations for variables, Study 1.

	Variable	*M*	*SD*	1	2	3	4	5
1	Conspiratorial belief	2.35	1.08					
2	Interdependent self‐construal	5.19	.94	.21***				
3	Vaccine acceptance	5.91	1.47	−.47***	.07			
4	Vaccination status	2.57	.77	−.38***	−.09	.63***		
5	Age	32.81	8.58	−.08	−.10	.04	−.01	
6	Gender	–	–	−.22***	−.29***	−.004	.09	−.09

*Note*: *N* = 290. Vaccination status (1 = Have not been vaccinated even though the vaccine had been available, 2 = Have been partially vaccinated, 3 = Have been fully vaccinated). Gender (0 = Man, 1 = Woman). We excluded three participants who did not identify as a man or a woman for the row of gender. ***p < .001.

**TABLE 2:** Results of regression analyses predicting vaccine acceptance and vaccine status, Study 1.

	Vaccine acceptance			Vaccine status		
	*b* (*SE*)	*t*	*β*	*b* (*SE*)	*t*	*β*
*Predictors*						
Intercept	5.83 (.07)	79.33***	.05	−1.88 (.20)	−9.24***	−.88
Conspiratorial beliefs (CB)	−.72 (.07)	−10.56***	−.53	−.92 (.15)	−6.04***	−.46
Interdependent self‐construal (ISC)	.30 (.08)	3.86***	.19	−.24 (.19)	−1.26	−.11
CB × ISC	.35 (.07)	4.92***	.24	.39 (.17)	2.33*	.19
*Conditional effects*						
High ISC (+1 SD)	−.40 (.09)	−4.32***	−.29	−.55 (.18)	−3.03**	
Low ISC (−1 SD)	−1.05 (.10)	−10.59***	−.77	−1.28 (.25)	−5.16***	
*R* ^2^	.31			.27		

*Note*: *N* = 290. *b*s are unstandardized coefficients. *β*s are standardized coefficients. Conspiratorial beliefs and interdependent self‐construal were mean‐centred. *p < .05; **p < .01; ***p < .001.

#### Vaccine acceptance

**FIGURE 2:**
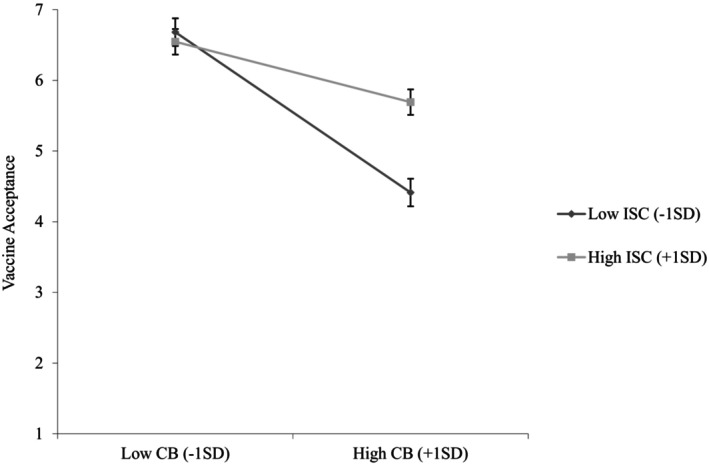
Vaccine acceptance as a function of conspiratorial beliefs and interdependent self‐construal (Study 1). CB, Conspiratorial beliefs; ISC, Interdependent self‐construal. 95% Confidence interval error bars were added to the graph.

We found that conspiratorial beliefs were negatively associated with vaccine acceptance (*b* = −.72, *SE* = .07, *p <* .001, 95%CI [−.86, −.59]), supporting H1. We also found that interdependent self‐construal was positively associated with vaccine acceptance (*b* = .30, *SE* = .08, *p* < .001, 95%CI [.15, .46]). In addition, a significant interactive effect of conspiratorial beliefs and interdependent self‐construal on vaccine acceptance emerged (*b* = .35, *SE* = .07, *p* < .001, 95%CI [.21, .49]). Specifically, the negative relationship between conspiratorial beliefs and vaccine acceptance was stronger when interdependent self‐construal was low (1 standard deviation below the mean; –1 SD; *b* = −1.05, *SE* = .10, *p* < .001, 95%CI [−1.25, −.86]) than when it was high (1 standard deviation above the mean; +1 SD; *b* = −.40, *SE* = .09, *p <* .001, 95%CI [−.58, −.22]; see Figure 2), supporting H3.

#### Vaccination status

We conducted an ordinal logistic regression analysis in Mplus version 8.0 (Muthén & Muthén, 2012) to examine the effects of conspiratorial beliefs and interdependent self‐construal on vaccination status. We found that conspiratorial beliefs were negatively associated with the likelihood of vaccination (*b* = −.92, *SE* = .15, *Z* = −6.04, *p < *.001, 95%CI [−1.21, −.62]), supporting H1. We also found an interactive effect of conspiratorial beliefs and interdependent self‐construal on the likelihood of vaccination (*b* = .39, *SE* = .17, *Z* = 2.33, *p* = .020, 95%CI [.06, .72]). The relationship between conspiratorial beliefs and the likelihood of vaccination was weaker when interdependent self‐construal was high (+1 SD; *b* = −.55, *SE* = .18, *Z* = −3.03, *p* = .002, 95%CI [−.92, −.20]) than when it was low (−1 SD; *b* = −1.28, *SE* = .25, *Z* = −5.16, *p <* .001, 95%CI [−1.77, −.81]), supporting H3.

### Discussion

Study 1 found that conspiratorial beliefs were negatively associated with vaccine acceptance and self‐reported vaccination status; further, this effect was attenuated among individuals whose interdependent self‐construal was more accessible.

## STUDY 2

As the world moves beyond the COVID‐19 lockdown era, new variants of the virus continue to pose risks (Blum, 2024), and health officials continue to urge individuals to get vaccinated (COVID‐19 Workforce Impacts in the US | McKinsey, n.d.; Abdollahi et al., 2022; Lundberg‐Morris et al., 2023). Study 2 aimed to expand our exploration to examine the acceptance of updated COVID‐19 vaccines (H1 and H3) and prosocial motivation as the key mediating mechanism (H2, H4 and H5).

### Participants and procedure

Participants were 643 US residents recruited on Prolific in March 2024, who received $2.00 for completing the study (gender: 49.8% men, 48.1% women, 1.7% non‐binary individuals, 0.4% who preferred not to disclose; *M*_*age*_ = 38.8 years, *SD*_*age*_ = 11.35; education: 10.3% had some high school education or a high school degree, 11.8% had an associate degree, 18.5% had some college education, 41.5% had an undergraduate degree, 17.9% had a graduate degree; race: 70.8% Caucasian, 13.8% Black, 5.9% Hispanic, 7.6% Asian, 1.9% other).

### Measures

Conspiratorial beliefs (*α* = .87) and interdependent self‐construal (*α* = .70) were measured using the same items as in Study 1.

#### Prosocial motivation

Participants responded to a four‐item measure of prosocial motivation developed by Bendell (2017) (e.g. ‘I care about benefiting others’, 1 = *strongly disagree*, 7 = *strongly agree*; *α* = .92).

#### Updated vaccine acceptance

The updated three‐item vaccine acceptance measure was adapted from Study 1, for example, ‘I believe a COVID‐19 booster is important in preventing the spread of COVID‐19’ (*α* = .96).

#### Control variables

As in Study 1, we measured and controlled for participants' independent self‐construal (*α* = .78). We also controlled for age, gender, race, education level, political affiliation and vaccination status, as these variables may affect their attitudes towards vaccination. The pattern of results remained consistent with or without these control variables. We report results without controls below and results with controls in Appendix S1.

### Results

**FIGURE 3:**
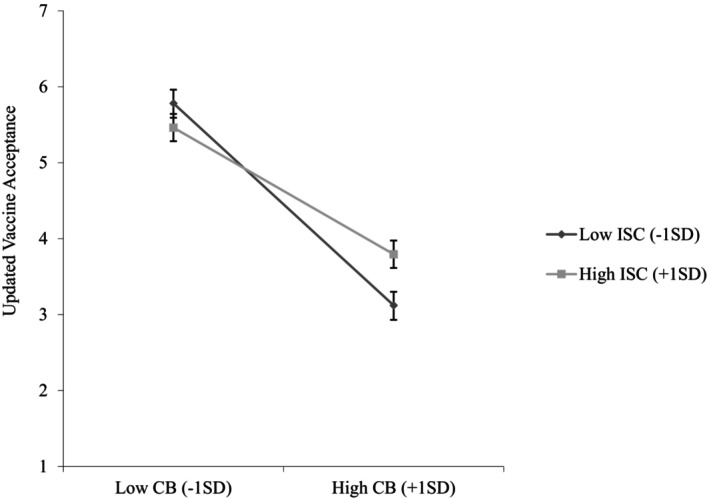
Updated vaccine acceptance as a function of conspiratorial beliefs and interdependent self‐construal (Study 2). CB, Conspiratorial beliefs; ISC, Interdependent self‐construal. 95% Confidence interval error bars were added to the graph.

Table 3 shows Study 2 means, standard deviations and correlations. We mean‐centred conspiratorial beliefs and interdependent self‐construal and used the same analyses as in Study 1. The results of the regression analyses are reported below and in Table 4. Conspiratorial beliefs were negatively associated with updated vaccine acceptance (*b* = −1.12, *SE* = .07, *p <* .001, 95%CI [−1.26, −.98]), replicating prior findings and supporting H1. The effect of interdependent self‐construal on updated vaccine acceptance was not significant (*b* = .10, *SE* = .07, *p* = .193, 95%CI [−.05, .24]). However, the predicted interaction between conspiratorial beliefs and interdependent self‐construal was significant (*b* = .28, *SE* = .07, *p <* .001, 95%CI [.15, .41]). Conspiratorial beliefs had a stronger association with updated vaccine acceptance when interdependent self‐construal was low (−1 SD; *b* = −1.37, *SE* = .09, *p <* .001, 95%CI [−1.56, −1.19]) than when it was high (+1 SD; *b* = −.86, *SE* = .09, *p <* .001, 95%CI [−1.04, −.68]; see Figure 3), supporting H3.

**TABLE 3:** Means, standard deviations and correlations for variables, Study 2.

	Variable	*M*	*SD*	1	2	3	4	5
1	Conspiratorial beliefs	2.10	.97					
2	Interdependent self‐construal	4.54	.93	.08*				
3	Prosocial motivation	5.79	1.00	−.10**	.34***			
4	Updated vaccine acceptance	4.56	2.03	−.52***	.01	.16***		
5	Age	38.77	11.35	−.02	−.03	.04	−.02	
6	Gender	–	–	.004	−.02	.10**	.03	.12**

*Note*: *N* = 643. Gender (0 = Man, 1 = Woman). We excluded 14 participants who did not identify as a man or a woman for the row of gender. *p < .05; **p < .01; ***p < .001.

**TABLE 4:** Results of regression analyses predicting updated vaccine acceptance, Study 2.

	Prosocial motivation			Updated vaccine acceptance		
	*b* (*SE*)	*t*	*β*	*b* (*SE*)	*t*	*β*
*Predictors*						
Intercept	5.78 (.04)	157.61***	−.01	3.35 (.40)	8.30***	.00
CB	−.14 (.04)	−3.63***	−.13	−1.08 (.07)	−15.22***	−.51
ISC	.37 (.04)	9.29***	.34			
CB × ISC	.08 (.04)	2.10*	.07			
Prosocial motivation				.21 (.07)	3.05**	.10
*Conditional effects*						
High ISC (+1 SD)	−.07 (.05)	−1.38	−.07			
Low ISC (−1 SD)	−.21 (.05)	−4.03***	−.20			

	*b* (SE)	95%CI	
*Conditional indirect effects*			
High ISC (+1 SD)	−.01 (.01)	[−.04, .004]	
Low ISC (−1 SD)	−.04 (.02)	[−.10, −.01]	
*R* ^2^	.14		.28

*Note*: *N* = 643. *b*s are unstandardized coefficients. *β*s are standardized coefficients. Conspiratorial beliefs and interdependent self‐construal were mean‐centred. *p < .05; **p < .01; ***p < .001.

Abbreviations: CB, Conspiratorial beliefs; ISC, Interdependent self‐construal.

To test our proposed moderated mediation model, we ran Hayes's (2017) SPSS PROCESS macro Model 7 with 5000 bootstrapped samples and a 95% confidence interval using prosocial motivation as the mediator. We found that conspiratorial beliefs were negatively associated with prosocial motivation (*b* = −.14, *SE* = .04, *p <* .001, 95%CI [−.21, −.06]) and prosocial motivation was positively related to updated vaccine acceptance (*b* = .21, *SE* = .07, *p* = .002, 95%CI [.07, .34]). Furthermore, prosocial motivation mediated the negative relationship between conspiratorial beliefs and updated vaccine acceptance (*IE* = −.03, *SE* = .01, 95%CI [−.06, −.01]), supporting H2.

**FIGURE 4:**
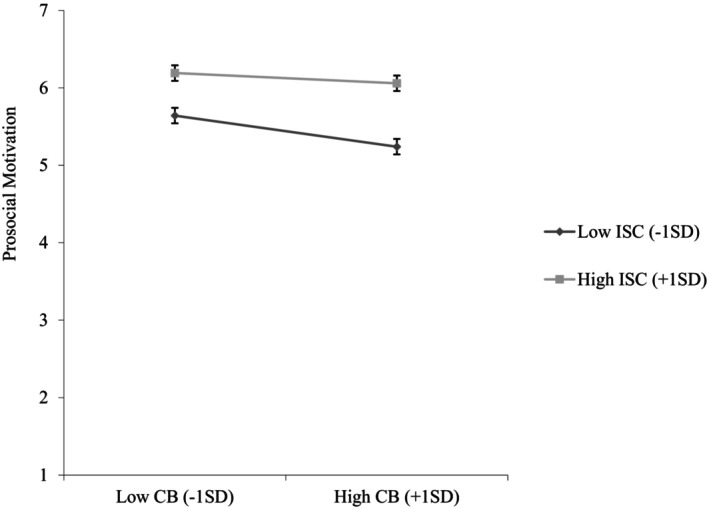
Prosocial motivation as a function of conspiratorial beliefs and interdependent self‐construal (Study 2). CB, Conspiratorial beliefs; ISC, Interdependent self‐construal. 95% Confidence interval error bars were added to the graph.

Furthermore, interdependent self‐construal was positively associated with prosocial motivation (*b* = .37, *SE* = .04, *p <* .001, 95%CI [.29, .45]) and moderated the relationship between conspiratorial beliefs and prosocial motivation (*b* = .08, *SE* = .04, *p* = .036, 95%CI [.01, .15]). Conspiratorial beliefs were negatively associated with prosocial motivation only when interdependent self‐construal was low (−1 SD; *b* = −.21, *SE* = .05, *p* < .001, 95%CI [−.31, −.11]), but not when interdependent self‐construal was high (+1 SD; *b* = −.07, *SE* = .05, *p* = .168, 95%CI [−.17, .03]; see Figure 4), supporting H4.

Finally, we found that interdependent self‐construal moderated the indirect relationship between conspiratorial beliefs and updated vaccine acceptance through prosocial motivation. In particular, conspiratorial beliefs were negatively associated with updated vaccine acceptance via prosocial motivation (−1 SD; *IE* = −.04, *SE* = .02, 95%CI [−.10, −.01]) when interdependent self‐construal was low. This effect was not significant when interdependent self‐construal was high (+1 SD; *IE* = −.01, *SE* = .01, 95%CI [−.04, .004]), supporting H5.

### Discussion

These results added to Study 1's findings by showing that conspiratorial beliefs were negatively associated with updated vaccine acceptance, which is important for maintaining continual protection against the disease. We also showed that reduced prosocial motivation underlies this negative effect. Finally, we confirmed that interdependent self‐construal accessibility weakened both the direct effect of conspiratorial beliefs on vaccine acceptance and the indirect effect through prosocial motivation.

## STUDY 3

Study 2 provided support for H1 through H5. In Study 3, we further examined H1 and H3 by collecting data from India, the United Kingdom and the United States. We extended our data collection beyond United States‐based samples in line with recommendations to test the generalizability of findings across different cultural contexts (Henrich et al., 2010).

Past work suggests that individuals from India possess higher levels of interdependent self‐construal than those from Western cultures such as the United Kingdom and the United States (Kitayama et al., 1990; Markus & Kitayama, 1991). Accordingly, Indian participants may exhibit behaviours similar to the participants in Studies 1 and 2 with more accessible interdependent self‐construal (i.e. +1 SD), whereas participants from the United Kingdom and the United States may demonstrate behaviours similar to the participants in Studies 1 and 2 with less accessible interdependent self‐construal (i.e. –1 SD). This suggests that the negative relationship between conspiratorial beliefs and vaccine acceptance would be weaker for individuals from India compared to those from the United Kingdom and the United States.

**FIGURE 5:**
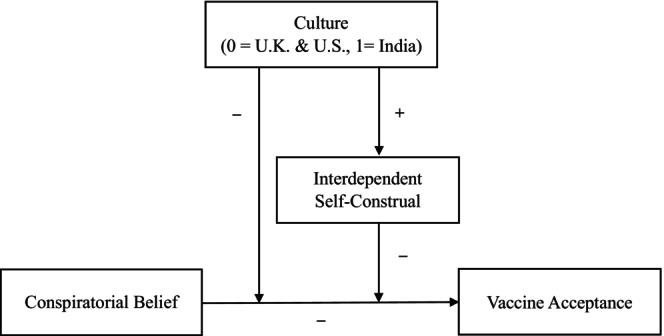
Theoretical model (Study 3).

We further predicted that the moderating effect of culture is mediated by interdependent self‐construal. Specifically, we expected our Indian participants to exhibit higher levels of interdependent self‐construal compared to participants from the United Kingdom and the United States. In line with H3, which predicts that interdependent self‐construal weakens the negative relationship between conspiratorial beliefs and vaccine acceptance, we hypothesized that compared to the UK and US participants, Indian participants' conspiratorial beliefs will have a weaker association with reduced vaccine acceptance (Figure 5).

### Participants and procedure

In September 2021, we recruited participants in the United States and India on Amazon's Mechanical Turk Prime (MTurk) and in the United Kingdom on Prolific. The participants were 487 adults (see Table 5 for demographic information by country) compensated $1.50.

**TABLE 5:** Demographics by country, Study 3.

Country	*N*	Age		Race (%)						Gender (%)		
		*M*	*SD*	Caucasian	East Asian	Hispanic	South Asian	Black	Other	Men	Women	Other
United States	156	39.20	10.85	79.5	4.5	4.5	1.9	8.3	1.3	55.8	43.6	.6
United Kingdom	180	33.92	10.10	80.0	2.8	.6	3.9	5.6	7.1	39.4	60.6	0
India	151	29.13	4.43	.7	6.0	2.6	90.1	0	.6	69.5	30.5	0

### Measures

Conspiratorial beliefs (*α* = .91), interdependent self‐construal (*α* = .79) and vaccine acceptance (*α* = .94) were measured using the same items as in previous studies.

#### Control variables

We controlled for participants' age, gender and educational level, as these variables may affect vaccination attitudes. The results remained significant with or without these control variables. We report results without controls below and results with controls in Appendix S1.

### Results

Table 6 shows Study 3 means, standard deviations and correlations. For means, standard deviations and correlations by country, please see the Appendix S1. We followed Grant and Wrzesniewski's (2010) process and conducted multiple linear regression analyses (see Table 7) using Mplus version 8.0 to test for mediated moderation – whether the moderating effect of culture (0 = United Kingdom and United States, 1 = India) might be attributable to differences in interdependent self‐construal. We used bias‐corrected confidence intervals with 5000 random samples (Stine, 1989).

**TABLE 6:** Means, standard deviations and correlations for variables, Study 3.

	Variable	*M*	*SD*	1	2	3	4	5
1	Culture	–	–					
2	Conspiratorial belief	2.71	1.26	.64***				
3	Interdependent self‐construal	5.02	1.06	.37***	.40***			
4	Vaccine acceptance	5.68	1.59	.07	−.20***	.32***		
5	Age	34.13	9.87	−.34***	−.22***	−.19***	−.07	
6	Gender	–	–	−.21***	−.10*	−.01	−.06	.06

*Note*: *N* = 487. Culture (0 = United Kingdom and United States, 1 = India). Gender (0 = Man, 1 = Woman). We excluded one participant who did not identify as a man or a woman for the row of gender. *p < .05; ***p < .001.

**TABLE 7:** Results of mediated moderation analysis, Study 3.

	ISC			Vaccine acceptance		
	*b* (*SE*)	*t*	*β*	*b* (*SE*)	*t*	*β*
*Predictors*						
Intercept	−.26 (.05)	−4.86***	−.25	5.26 (.10)	50.85***	3.23
CB				−.81 (.08)	−10.06***	−.62
Culture	.85 (.10)	8.59***	.37	−.07 (.28)	−.26	−.02
ISC				.61 (.09)	7.14***	.40
CB × Culture				.77 (.22)	3.50***	.33
CB × ISC				.29 (.06)	4.66***	.23
*Conditional effect*						
United Kingdom and United States				−.81 (.08)	−10.06***	
India				−.04 (.21)	−.17	
*R* ^2^	.14			.39		

*Note*: *N* = 487. Culture (0 = United Kingdom and United States, 1 = India). Conspiratorial beliefs and interdependent self‐construal were mean‐centred. *b*s are unstandardized coefficients. *β*s are standardized coefficients. ***p < .001.

Abbreviations: CB, Conspiratorial beliefs; ISC, Interdependent self‐construal.

**FIGURE 6:**
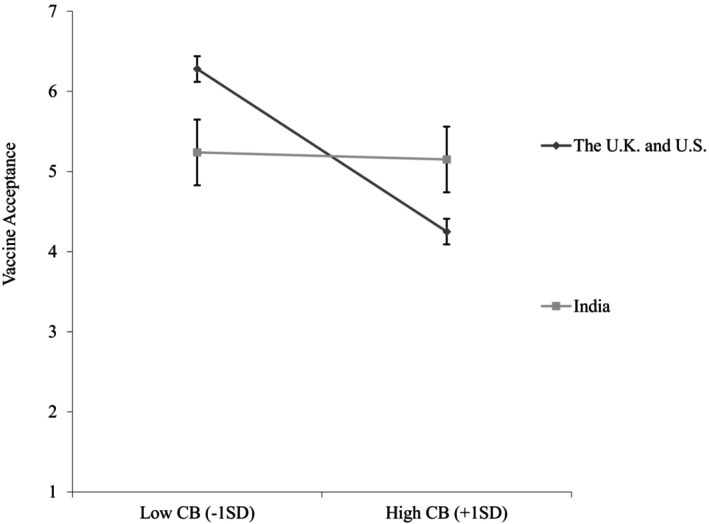
Vaccine acceptance as a function of conspiratorial beliefs and culture (Study 3). CB, Conspiratorial beliefs. 95% Confidence interval error bars were added to the graph.

As expected, conspiratorial beliefs were negatively associated with vaccine acceptance (*b* = −.81, *SE* = .08, *p* < .001, 95%CI [−.97, −.65]), supporting H1. Moreover, we found a significant interactive effect of conspiratorial beliefs and culture on vaccine acceptance (*b* = .77, *SE* = .22, *p* < .001, 95%CI [.32, 1.19]; see Figure 6). For participants from the United Kingdom and the United States, conspiratorial beliefs were negatively associated with vaccine acceptance (*b* = −.81, *SE* = .08, *p* < .001, 95%CI [−.97, −.65]), whereas the effect was not significant (*b* = −.04, *SE* = .21, *p* = .862, 95%CI [−.46, .36]) for participants from India. That is, the negative relationship between conspiratorial beliefs and vaccine acceptance is weaker for individuals from India compared to those from the United Kingdom and the United States.

Moreover, Indian participants exhibited higher levels of interdependent self‐construal than participants from the United Kingdom and United States (*b* = .85, *SE* = .10, *p* < .001, 95%CI [.64, 1.03]). In addition, a significant interactive effect of conspiratorial beliefs and interdependent self‐construal on vaccine acceptance emerged (*b* = .29, *SE* = .06, *p* < .001, 95%CI [.17, .41]). Specifically, the negative relationship between conspiratorial beliefs and vaccine acceptance was stronger when interdependent self‐construal was low (−1 SD; *b* = −1.11, *SE* = .11, *p* < .001, 95%CI [−1.31, −.87]) than when it was high (+1 SD; *b* = −.50, *SE* = .10, *p <* .001, 95%CI [−.73, −.33]), in line with H3. Moreover, we found that the indirect effect differed from zero (*b* = .24, *SE* = .07, 95%CI [.13, .39]), suggesting that interdependent self‐construal mediated the moderating effect of culture on the relationship between conspiratorial beliefs and vaccine acceptance.

### Discussion

These results confirmed that the relationship between conspiratorial beliefs and vaccine acceptance is moderated by culture, and interdependent self‐construal underlies the moderating role of culture. By collecting data in India, the United Kingdom and the United States, we were able to examine our hypotheses across different cultural contexts.

## STUDY 4

One objective of Study 4 was to further demonstrate the robustness of our findings (H1 and H3). A limitation of Studies 1, 2 and 3 is that interdependent self‐construal was measured. Hence, the observed relationships were correlational in nature. In this study, we manipulated interdependent self‐construal to garner more confidence in the causal role of interdependent self‐construal in mitigating the negative impact of conspiratorial beliefs on vaccine acceptance.

### Participants and procedure

Participants were 702 US residents recruited on MTurk in September 2022 who were compensated $1.50 for completing the study (gender: 49.6% men, 49.4% women, 0.4% non‐binary individuals, 0.6% who preferred not to disclose; *M*_*age*_ = 41.85 years, *SD*_*age*_ = 12.72; education: 9.8% had some high school education or a high school degree, 8.8% had an associate degree, 17.7% had some college education, 44.9% had an undergraduate degree, 18.8% had a graduate degree; race: 77.5% Caucasian, 9.7% Black, 4.1% Hispanic, 6.5% Asian, 2.2% other). Participants were randomly assigned to one of two self‐construal conditions (interdependent vs. independent).

### Manipulation

We manipulated the salience of self‐construal following methods adapted from Maddux et al. (2010). In the *interdependent self‐construal condition*, participants wrote a brief essay about their friendships and camaraderie with others and how they might foster these relationships. In the *independent self‐construal condition*, participants wrote about their unique character and skills and how they might stand out relative to others (see the Appendix S1 for full details). Participants then responded to our key measures.

### Measures

Conspiratorial beliefs (*α* = .91) and vaccine acceptance (*α* = .95) were measured using the same items as in the previous studies.

#### Control variables

Although participants were randomly assigned to a self‐construal condition, we controlled for participants' age, gender, race, educational level, political affiliation and vaccination status. The pattern of results remained the same with or without these control variables. We report results without controls below and results with controls in Appendix S1.

### Results

Table 8 shows Study 4 means, standard deviations and correlations.

**TABLE 8:** Means, standard deviations and correlations for variables, Study 4.

	Variable	*M*	*SD*	1	2	3	4
1	Conspiratorial beliefs	2.11	1.09				
2	Self‐construal manipulation	–	–	.02			
3	Vaccine acceptance	5.19	1.96	−.54***	.002		
4	Age	41.85	12.72	−.04	−.03	−.004	
5	Gender	–	–	.06	.01	−.04	.12**

*Note*: *N* = 702. Self‐construal manipulation (0 = Independent self‐construal condition, 1 = Interdependent self‐construal condition). Gender (0 = Man, 1 = Woman). We excluded seven participants who did not identify as a man or a woman for the row of gender. **p < .01; ***p < .001.

#### Manipulation check

Two individuals blind to the conditions independently coded participants' self‐construal essays to assess how much the responses emphasized their interdependence and independence (1 = *not at all*, 7 = *a lot*). The coders exhibited significant agreement in their coding of both interdependent self‐construal (ICC(1) = .75, *F* = 4.03, *p* < .001) and independent self‐construal (ICC(1) = .85, *F* = 6.54, *p* < .001). The mean scores across the two coders were used as our manipulation check measure.

As expected, participants in the interdependent self‐construal condition reported higher levels of interdependent self‐construal (*M* = 4.23, *SD* = 1.74) than those in the independent self‐construal condition (*M* = 1.82, *SD* = 1.00), *t*(675) = 22.13, *p <* .001, *d* = 1.70. They also reported lower levels of independent self‐construal (*M* = 2.76, *SD* = 1.57) than those in the independent self‐construal condition (*M* = 6.14, *SD* = 1.07), *t*(675) = −32.82, *p* < .001, *d* = −2.53. This suggests that the self‐construal manipulation was successful.

#### Test of hypotheses

**FIGURE 7:**
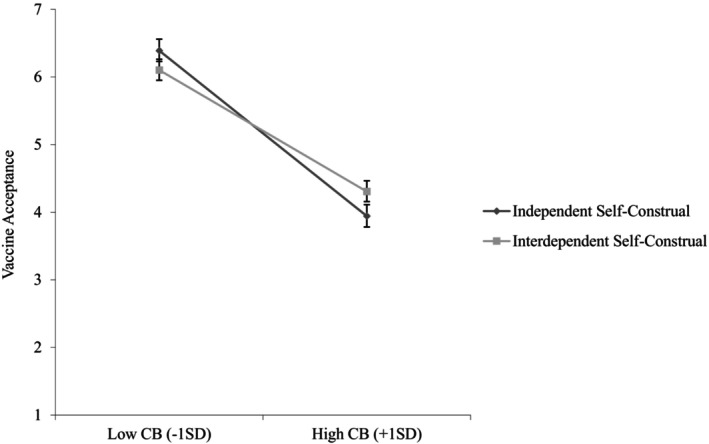
Vaccine acceptance as a function of conspiratorial beliefs and interdependent self‐construal manipulation (Study 4). CB, Conspiratorial beliefs. 95% Confidence interval error bars were added to the graph.

Using procedures similar to those in the previous studies, we mean‐centred conspiratorial beliefs. The results of the regression analyses are reported below and in Table 9. We found that conspiratorial beliefs were negatively associated with vaccine acceptance (*b* = −1.13, *SE* = .08, *p* < .001, 95%CI [−1.29, −.96]), supporting H1. The effect of the self‐construal manipulation on vaccine acceptance was not significant (*b* = .04, *SE* = .12, *p* = .758, 95%CI [−.21, .28]). Central to this research, the predicted interaction between conspiratorial beliefs and self‐construal manipulation was significant (*b* = .30, *SE* = .11, *p* = .009, 95%CI [.07, .52]). Conspiratorial beliefs were negatively associated with vaccine acceptance in the independent self‐construal condition (*b* = −1.13, *SE* = .08, *p* < .001, 95%CI [−1.29, −.96]). However, this relationship was weaker in the interdependent self‐construal condition (*b* = −.83, *SE* = .08, *p* < .001, 95%CI [−.98, −.67]; see Figure 7), supporting H3.

**TABLE 9:** Results of regression analyses predicting vaccine acceptance and vaccine status, study 4.

	Vaccine acceptance		
	*b* (*SE*)	*t*	*β*
*Predictors*			
Intercept	5.17 (.09)	59.13***	−.01
CB	−1.13 (.08)	−13.64***	−.63
SCM	.04 (.12)	.31	.02
CB × SCM	.30 (.11)	2.62**	.17
*Conditional effects*			
Interdependent self‐construal condition	−.83 (.08)	−10.44***	−.46
Independent self‐construal condition	−1.13 (.08)	−13.64***	−.63
*R* ^2^	.30		

*Note*: *N* = 702. *b*s are unstandardized coefficients. *β*s are standardized coefficients. Conspiratorial beliefs were mean‐centred. **p < .01; ***p < .001.

Abbreviations: CB, Conspiratorial beliefs; SCM, Self‐construal manipulation (0 = Independent self‐construal condition, 1 = Interdependent self‐construal condition).

### Discussion

Study 4 replicated the moderating role of interdependent self‐construal on the effect of conspiratorial beliefs on vaccine acceptance. By manipulating the accessibility of independent versus interdependent self‐construal, we presented evidence for the causal role of interdependent self‐construal as a moderator.

## STUDY 5

Our pre‐registered Study 5 introduced several improvements over the previous studies. First, to extend our generalizability and broaden our findings beyond COVID‐19, Study 5 examined our hypotheses in the context of a fictitious virus and associated conspiracy theories. Second, we manipulated conspiratorial beliefs to more clearly establish causality in examining H1 and H3. Third, Study 4 found differences between interdependent and independent self‐construal but left unclear whether the effects were driven by interdependent self‐construal suppressing or independent self‐construal strengthening the link between conspiratorial beliefs and vaccine acceptance. Therefore, this study sought to address this concern by comparing an interdependent self‐construal condition to a baseline condition. Finally, as in Study 2, we examined prosocial motivation as the key mediating mechanism (H2, H4 and H5) by coding prosocial motivation mentions in essays written by participants discussing their health concerns related to the fictitious virus.

### Participants and procedure

Participants were 692 US residents of Prolific who were compensated $1.50 for completing the study in August 2024. The actual number of participants surveyed was slightly lower than the planned recruitment goal of 700 individuals because some participants did not complete the study. Consistent with our pre‐registration, we excluded 135 participants who did not follow the manipulation instructions (e.g. did not respond, did not answer the question or cut‐and‐pasted responses from elsewhere instead of writing). These exclusions are consistent with those found in research conducted by Fairlamb et al. (2022) and Tsai and Zeng (2021). Our final sample size was 557 participants (gender: 35.5% men, 63.4% women, 0.9% non‐binary individuals, 0.4% who preferred not to disclose; *M*_*age*_ = 40.58 years, *SD*_*age*_ = 11.91; education: 14.3% had some high school education or a high school degree, 10.1% had an associate degree, 19.1% had some college education, 34.0% had an undergraduate degree, 22.5% had a graduate degree; race: 73.2% Caucasian, 17.1% Black, 3.6%, Hispanic, 4.3% Asian, 1.8% other).

We first manipulated conspiratorial beliefs to explore their effect on reduced vaccine acceptance using a paradigm inspired by past research (Cookson et al., 2021; Nera et al., 2024; van Prooijen et al., 2018), which focused on a new disease. Participants then answered the conspiratorial beliefs manipulation check questions and were directed to the self‐construal manipulation (Gardner et al., 1999). Finally, participants assessed their levels of vaccine acceptance, and to measure prosocial motivation, they wrote an essay about the virus, discussing their health concerns.

### Conspiratorial beliefs manipulation

Participants were asked to imagine going online where they encountered an article on the spread of a new disease, Dyspeptmeria, which stated that ‘Infected individuals have been found on at least three continents, with those infected suffering from respiratory and digestive issues’ (see the Appendix S1 for full details).

In the *low conspiratorial beliefs* condition, participants read an article that did not suggest the presence of conspiracy theories. For example, participants read that the evidence of the origins of the virus was trustworthy and robust, with scientists agreeing ‘that the disease likely originated from contact between bats and humans at a bat sanctuary in Russia … As the virus continues to spread across the U.S., many believe that media concerns about the dangers of Dyspeptmeria have been validated’.

In the *high conspiratorial beliefs* condition, participants read an article that suggested the presence of conspiracy theories. For instance, participants read that ‘While scientists claim that the disease likely originated from contact between bats and humans at a bat sanctuary in Russia, some online discussions have found the scientists' evidence and conclusions weak, suggesting that information is being deliberately held back from the public … As the virus continues to spread across the U.S., many believe that the media is exaggerating its severity for their own purposes’.

### Interdependent self‐construal manipulation

The interdependent self‐construal manipulation was adapted from Gardner et al. (1999). Participants were instructed to read a descriptive paragraph of approximately 100 words about a trip to the city and to highlight words of a particular type. In the *interdependent self‐construal* condition, the paragraph was written in first‐person plural, and participants were asked to highlight all pronouns (e.g. *we, us* and *our*). In the *baseline* condition, the paragraph was written in the third person, and participants were asked to highlight all nouns (e.g. *city, view, skyscraper*). There were 19 pronouns and nouns in each of the respective conditions.

### Measures

#### Conspiratorial beliefs manipulation check

Three items were adapted from the five‐item scale in the previous studies (e.g. ‘A lot of information about Dyspeptmeria is deliberately held back from the public’, *α* = .71). Two items that describe conspiracy theories irrelevant to the manipulation were dropped.

#### Prosocial motivation

Participants were asked to write a short essay reflecting on the article about Dyspeptmeria. They were prompted with the statement: ‘We would like to know how concerned you are about your own health and/or the health of others’ and were instructed to write at least three sentences describing their thoughts. The essays were submitted to the Linguistic Inquiry and Word Count (LIWC) text analysis programme (Pennebaker et al., 2001), which calculates the percentage of words in a written sample that fall within predefined categories. In line with previous research (Frimer et al., 2014; Nai et al., 2018; Ye et al., 2020), we employed the prosocial dictionary to calculate the proportion of prosocial words (e.g. care, helpful, selfless) that participants wrote relative to the total number of words.

#### Vaccine acceptance

The vaccine acceptance measure was adapted from previous studies and made applicable to the fictitious vaccine (e.g. ‘I believe a Dyspeptmeria vaccine is important in preventing the spread of Dyspeptmeria’, *α* = .96).

### Results

Table 10 shows Study 5 means, standard deviations and correlations.

**TABLE 10:** Means, standard deviations and correlations for variables, Study *5.*

	Variable	*M*	*SD*	1	2	3	4	5
1	Conspiratorial beliefs manipulation	–	–					
2	Self‐construal manipulation	–	–	.02				
3	Prosocial motivation	.49	1.10	−.08	.03			
4	Vaccine acceptance	4.75	1.81	−.05	−.02	.17***		
5	Age	40.58	11.91	.004	.004	.04	.001	
6	Gender	–	–	.04	−.02	.07	−.05	.14***

*Note*: *N* = 557. Conspiratorial beliefs manipulation (0 = Low conspiratorial beliefs condition, 1 = High conspiratorial beliefs condition). Self‐construal manipulation (0 = Baseline condition, 1 = Interdependent self‐construal condition). Gender (0 = Man, 1 = Woman). We excluded seven participants who did not identify as a man or a woman for the row of gender. ***p < .001.

#### Manipulation check

As expected, participants in the high conspiratorial beliefs condition reported higher levels of conspiratorial beliefs (*M* = 2.76, *SD* = .82) than those in the low conspiratorial beliefs condition (*M* = 2.15, *SD* = .87), *t*(554) = 8.53, *p* < .001, *d* = .72. This suggests that the manipulation of conspiratorial beliefs was successful.

#### Test of hypotheses

To test H1 and H3, we performed a 2 × 2 between‐subjects ANOVA using conspiratorial beliefs and interdependent self‐construal as fixed factors and vaccine acceptance as the dependent variable. Conspiratorial beliefs did not affect vaccine acceptance (*M*_high_ = 4.66, *SD* = 1.87; *M*_low_ = 4.85, *SD* = 1.75), *F*(1, 553) = 1.46, *p* = .227, *η* ^2^ = .003. Therefore, H1 was not supported. Interdependent self‐construal also did not affect vaccine acceptance (*M*_interdependent_ = 4.71, *SD* = 1.78; *M*_baseline_ = 4.79, *SD* = 1.85), *F*(1, 553) = .29, *p* = .590, *η* ^2^ = .001. The interactive effect of the conspiratorial beliefs and interdependent self‐construal on vaccine acceptance did not emerge, *F*(1, 553) = .24, *p* = .621, *η* ^2^ = .00. Therefore, H3 was not supported.

To test H2, which states that prosocial motivation mediates the relationship between conspiratorial beliefs and vaccine acceptance, we used Hayes's (2017) SPSS PROCESS macro Model 4 using 5000 bootstrapped samples. We found that conspiratorial beliefs did not significantly affect prosocial motivation (*M*_high_ = .41, *SD* = .91; *M*_low_ = .58, *SD* = 1.26; *b* = −.18, *SE* = .09, *p* = .059, 95%CI [−.36, .01]). However, prosocial motivation was positively related to vaccine acceptance (*b* = .27, *SE* = .07, *p* < .001, 95%CI [.13, .41]). Prosocial motivation did not mediate the relationship between conspiratorial beliefs and vaccine acceptance (*b* = −.05, *SE* = .03, 95%CI [−.11, .001]).

**FIGURE 8:**
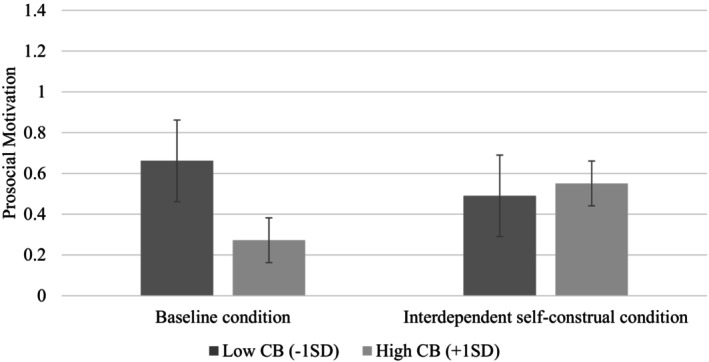
Prosocial motivation as a function of conspiratorial beliefs manipulation and interdependent self‐construal manipulation (Study 5). CB, Conspiratorial beliefs. 95% Confidence interval error bars were added to the graph.

To test H4, which states that interdependent self‐construal weakens the negative relationship between conspiratorial beliefs and prosocial motivation, we performed a two‐way ANOVA, with conspiratorial beliefs and interdependent self‐construal as fixed factors and prosocial motivation as the dependent variable. An interactive effect of conspiratorial beliefs and interdependent self‐construal on prosocial motivation emerged (*F*(1, 553) = 5.56, *p* = .019, *η* ^2^ = .01). We conducted pairwise comparisons and found that for participants in the baseline condition, conspiratorial beliefs reduced prosocial motivation (*M*_high_ = .26, *SD* = .69; *M*_low_ = .66, *SD* = 1.32), *t*(553) = 3.02, *p* = .003. However, for participants in the interdependent self‐construal condition, conspiratorial beliefs did not affect levels of prosocial motivation (*M*_high_ = .54, *SD* = 1.06; *M*_low_ = .50, *SD* = 1.20), *t*(553) = −.30, *p* = .763 (see Figure 8), supporting H4.

To test H5, we used the same method as in Study 2 (SPSS PROCESS macro Model 7 with 5000 bootstrapped samples and a 95% confidence interval). The results of the regression analyses are reported below and in Table 11. Interdependent self‐construal moderated the indirect relationship between conspiratorial beliefs and vaccine acceptance via prosocial motivation. The indirect effect was negative and significant (*b* = −.11, *SE* = .04, 95%CI [−.19, −.04]) in the baseline condition, but not in the interdependent self‐construal condition (*b* = .01, *SE* = .04, 95%CI [−.07, .09]), supporting H5.

**TABLE 11:** Results of regression analyses predicting vaccine acceptance, Study 5.

	Prosocial motivation			Vaccine acceptance		
	*b* (*SE*)	*t*	*β*	*b* (*SE*)	*t*	*β*
*Predictors*						
Intercept	.66 (.09)	7.06***	.19	4.69 (.12)	40.46***	.05
CBM	−.40 (.13)	−3.02**	−.37	−.14 (.15)	−.91	−.08
SCM	−.16 (.13)	−1.20	−.15			
CBM × SCM	.44 (.18)	2.36*	.41			
Prosocial motivation				.27 (.07)	3.90***	.16
*Conditional effects*						
Interdependent condition	.04 (.13)	.30	.04			
Baseline condition	−.40 (.13)	−3.02***	−.37			

	*b* (*SE*)	95%CI	
*Conditional indirect effects*			
Interdependent condition	.01 (.04)	[−.07, .09]	
Baseline condition	−.11 (.04)	[−.19, −.04]	
*R* ^2^	.02		.03

*Note*: *N* = 557. *b*s are unstandardized coefficients. *β*s are standardized coefficients. *p < .05; **p < .01; ***p < .001.

Abbreviations: CBM, Conspiratorial beliefs manipulation (0 = Low conspiratorial beliefs condition, 1 = High conspiratorial beliefs condition); SCM, Self‐construal manipulation (0 = Baseline condition, 1 = Interdependent self‐construal condition).

### Discussion

Consistent with Study 2, Study 5 found that interdependent self‐construal moderated the effect of conspiratorial beliefs on prosocial motivation (H4) and the effect of conspiratorial beliefs on vaccine acceptance via prosocial motivation (H5). By comparing an interdependent self‐construal condition to a baseline condition, we provided evidence that our effect was driven by interdependent self‐construal and not by independent self‐construal. Notably, the predicted effects of conspiratorial beliefs (H1) and their interaction with interdependent self‐construal (H3) on vaccine acceptance did not emerge. Moreover, the mediating role of prosocial motivation in the relationship between conspiratorial beliefs and vaccine acceptance (H2) did not emerge. We elaborate on these results in the General Discussion.

## GENERAL DISCUSSION

This research documents the consequential role that conspiratorial beliefs play in vaccine acceptance and introduces a novel intervention to address this issue. Four studies demonstrated that conspiratorial beliefs were negatively associated with vaccine acceptance, with this relationship being weaker among individuals whose interdependent self‐construal was more accessible, regardless of whether the construct was measured (Studies 1 through 3) or manipulated (Study 4). Moreover, Study 2 demonstrated that prosocial motivation mediated the negative relationship between conspiratorial beliefs and vaccine acceptance, and both Studies 2 and 5 found that interdependent self‐construal moderated this mediated relationship. Finally, Study 3 showed that compared to the UK and US participants, Indian participants' conspiratorial beliefs were more weakly associated with reduced vaccine acceptance, as consistent with their more accessible interdependent self‐construal.

### Theoretical implications

Our research makes significant contributions to the study of conspiratorial beliefs and misinformation. First, we advance theory by introducing an effective social intervention – increasing the accessibility of interdependent self‐construal – to combat the impact of conspiratorial beliefs. This approach differs from cognitive interventions, which may be more helpful in preventing the adoption of conspiratorial beliefs but have limited effectiveness in challenging pre‐existing beliefs (Dow et al., 2021). Our findings are significant because they offer a strategy that can effectively combat conspiratorial beliefs when they are already widespread, as is the case with COVID‐19 conspiratorial beliefs (Schaeffer, 2020), and potential future disease outbreaks and the associated conspiracy beliefs that might arise.

Our research introduces a new perspective – that fostering a more relational self‐concept can help counteract the negative health consequences associated with conspiratorial beliefs. Our findings are consistent with research demonstrating that more collectivistic countries (Lu et al., 2021) or those with stronger social norms (Gelfand et al., 2021) typically exhibit more effective and coordinated responses to the pandemic. Our proposed intervention aims to enhance people's concern for others despite their conspiratorial beliefs by making salient their interdependent self‐construal, a strategy particularly useful in promoting vaccine acceptance in less interdependent societies (e.g. the United Kingdom and the United States; Markus & Kitayama, 1991).

### Practical implications

According to an analysis by Amin and Cox (2021), the United States incurred $5.7 billion in avoidable COVID‐19‐related health care expenses between June and August of 2021 alone, primarily due to preventable hospitalizations among unvaccinated individuals. This placed enormous financial strain on governments, hospitals and public health initiatives. Conspiratorial beliefs contribute to these costs by reducing confidence in and compliance with public health advice from government agencies such as the CDC (Dow et al., 2022). Lower confidence in and non‐compliance with public health advice present a challenge in current and future pandemics. Indeed, pandemics have reliably spawned conspiracy theories, as with H1N1 influenza in 2009 (Smallpage et al., 2017), Ebola in 2014 (Earnshaw et al., 2019) and Zika in 2016 (Sharma et al., 2017). In addition, new viruses are increasingly likely to cause novel pandemics (Carlson et al., 2022; de Oliveira & Tegally, 2023). Unfortunately, new conspiracy theories spreading through generative artificial intelligence‐aided technology seem inevitable (News, 2024), although recent research suggests that AI could also be useful in reducing conspiratorial beliefs (Costello et al., 2024). The social intervention identified in the current research offers a practical approach to buffering against the harm of conspiratorial beliefs.

Our work indicates that it may be beneficial for organizations and institutions to design and incorporate interventions in their communications that enhance individuals' interdependent self‐construal, without targeting conspiratorial beliefs that might prompt resistance and reactance. These interventions could be introduced through subtle changes in the framing of public health messages. Marketing research has consistently shown that images and interpersonal interactions can invoke interdependent self‐construal by simply referencing community, family or using collective pronouns (e.g. an advertisement that urges, ‘Give your family a chance at great taste’; Aaker & Lee, 2001; Kwak et al., 2017; S. Lee & Pounders, 2019; Ma et al., 2014; Simpson et al., 2018). These insights could easily be applied to a public health setting. For example, instead of focusing on the personal health implications of vaccines, public health messaging could highlight connections to one's community (e.g. ‘Here's what you can do to keep America safe’; Wang & Lee, 2020). Subtle interventions can also be introduced in educational programmes for health care professionals, community leaders and family members to guide them in conversations with individuals who endorse conspiratorial beliefs. For example, doctors could be trained to use collective pronouns and focus on shared benefits when discussing vaccines. Importantly, even slight increases in vaccination rates resulting from these changes could bolster public health and reduce financial burdens faced by the health care industry.

### Limitations and future directions

While the findings support our hypotheses, some limitations are noted. Studies 1, 2 and 3 used cross‐sectional data, which could raise concerns about common method variance (CMV; Lindell & Whitney, 2001). Given the correlational nature of the data, the causal relationship may be attributed to other constructs not included in the model. Studies 4 and 5 mitigated this concern by manipulating interdependent self‐construal. Study 5 also manipulated conspiratorial beliefs and introduced a baseline condition for self‐construal manipulation; the results provide evidence for a causal relationship among conspiratorial beliefs, interdependent self‐construal and prosocial motivation.

In Study 5, neither conspiratorial beliefs nor their interaction with interdependent self‐construal influenced vaccine acceptance. This may be because the scenario, which involved an imaginary disease and associated conspiracy theories, did not fully engage participants. As a result, effects only emerged for the more proximate variable of prosocial motivation. Such scenarios are likely less engaging than encountering conspiracy theories through interpersonal connections, news sources and social media in the real world, which lend them greater depth and weight. This difference may also explain why the direct effect of vaccine acceptance and the mediating role of prosocial motivation did not emerge. Moreover, this non‐significant effect of conspiratorial beliefs on prosocial motivation may be attributed to the manipulation of interdependent self‐construal. Specifically, while conspiratorial beliefs reduced prosocial motivation in the baseline condition, this effect was absent in the interdependent self‐construal condition, thus obscuring the overall direct effect of conspiratorial beliefs on prosocial motivation.

Nonetheless, future research should further explore the role of prosocial motivation, as replicating this effect is essential before drawing firm conclusions. One potential avenue is to provide more vibrant experimental manipulations of conspiratorial beliefs related to real‐world diseases to help uncover these effects. However, researchers should remain mindful of the ethical risks of increasing participants' susceptibility to real‐world vaccine hesitancy.

Our research provides further evidence that social interventions may be helpful. Future research can investigate other ways to strengthen social connections to combat vaccine hesitancy. For example, those who remain unvaccinated may face social exclusion (Bor et al., 2023) or bouts of long COVID‐19, which reduce their ability to participate in everyday life. Such exclusions from broader public life might increase anxiety and reinforce individuals' affiliations with conspiracy theory communities (Nera et al., 2022). Highlighting opportunities for individuals to socially connect could be another strategy to combat the adverse effects of conspiratorial beliefs (Lee et al., 2022).

Future research could also explore how enhancing prosocial motivation may apply to other conspiratorial beliefs. For example, researchers have found that HIV‐related conspiratorial beliefs, which are widespread in some communities, affect prevention and treatment‐related behaviours (Bogart et al., 2010; Bogart & Thorburn, 2005). Given the similarities between HIV and COVID‐19 conspiratorial beliefs, our work may help address this challenge. In addition, our findings likely generalize to other situations where conspiratorial beliefs inhibit prosocial behaviours. For example, our intervention may also be effective in weakening the negative relationship between climate change conspiratorial beliefs and pro‐environmental policy support (Biddlestone et al., 2022).

## CONCLUSION

This paper explores an intervention to mitigate conspiratorial beliefs' negative impact on vaccine acceptance. Our findings show that conspiratorial beliefs reduce vaccine acceptance, a relationship mediated by reduced prosocial motivation. However, making salient one's interdependent self‐construal weakens these effects. We hope this work will encourage researchers to explore further social and interpersonal interventions to counter the negative impact of conspiratorial beliefs.

## AUTHOR CONTRIBUTIONS

**Yingli Deng:** Methodology; data curation; formal analysis; funding acquisition; project administration; writing – original draft; writing – review and editing. **Cynthia S. Wang:** Conceptualization; methodology; supervision; funding acquisition; project administration; writing – original draft; writing – review and editing. **Gloria Danqiao Cheng:** Formal analysis; writing – review and editing. **Jennifer A. Whitson:** Methodology; funding acquisition; writing – review and editing. **Benjamin J. Dow:** Writing – review and editing; funding acquisition. **Angela Y. Lee:** Writing – review and editing.

## CONFLICT OF INTEREST STATEMENT

We have no conflicts of interest to disclose.

## ETHICAL APPROVAL

The research presented in this paper adheres to all APA ethical standards.

## Supporting information


Appendix S1.

## Data Availability

The analysis code and data for variables described in the paper are available on the project's Open Science Framework page (https://osf.io/9baxk/?view_only=b6382f98de4d4d07af2571d46eec4972).

## Footnotes

^1^
Across studies, we included additional measures for exploratory purposes. Studies 1 and 3 measured cultural tightness, personal control, secondary control, relational mobility, and participant COVID‐19 infection rate estimates. Also, Study 3 measured situational conspiratorial beliefs, Study 4 measured trait independent and interdependent self‐construal, and Study 5 measured general thoughts about conspiracy theories.

^2^
We also measured participants' masking acceptance using three similar items, e.g., “During the pandemic, it has been very important to me to wear a mask” (1 = strongly disagree to 7 = strongly agree) in our Studies 1 and 3, as we reasoned that our hypotheses should apply to COVID‐19 response compliance in general. Indeed, the pattern of results on masking acceptance parallels that of vaccine acceptance and is reported in Appendix S1.

^3^
We coded individuals as fully vaccinated if they had received all recommended doses of a COVID‐19 vaccine (e.g., two doses of the Pfizer or Moderna vaccine, or one dose of the J&J vaccine) regardless of whether or not they received an updated vaccine (i.e., “booster”) shot.

^4^
As the distribution of vaccine acceptance is negatively skewed across our studies, the normality assumption of the residuals was violated. To address this concern, we employed a bootstrapping technique, which does not rely on the normality assumption. This approach helps provide more reliable parameter estimates (Hancock & Liu, 2012; Hayes et al., 2017).
