# Supplementary material for: The role of interdependent self‐construal in mitigating the effect of conspiratorial beliefs on vaccine acceptance
Source: Br J Soc Psychol. 2024 Dec 9;64(1):e12836. doi: 10.1111/bjso.12836 (PMC11627010; doi:10.1111/bjso.12836)
Supplement: Supplementary file 1 — Appendix S1. [file BJSO-64-0-s001.docx]

**Appendix**

**Study 1 Measures**

***COVID-19 conspiratorial beliefs***

Please rate how much you agree or disagree with each of the following statements. (*1=Strongly disagree 5=Strongly agree*)

1. A lot of information about COVID-19 is deliberately held back from the public.
2. There is a cure for COVID-19, but it is being withheld by the government.
3. COVID-19 was created and spread by a government organization.
4. The existence of COVID-19 is a hoax perpetuated by the media.
5. COVID-19 was purposefully created in, and released from, a biochemistry lab.

***Interdependent self-construal***

Please rate how much you agree or disagree with each of the following statements. (*1=Strongly disagree 7=Strongly agree*)

1. Even when I strongly disagree with group members, I avoid an argument.
2. I will sacrifice my self interest for the benefit of the group I am in.
3. I feel good when I cooperate with others.
4. I will stay in a group if they need me, even when I am not happy with the group.
5. It is important to me to respect decisions made by the group.

***Independent*** ***self-construal***

Please rate how much you agree or disagree with each of the following statements. (*1=Strongly disagree 7=Strongly agree*)

1. I enjoy being unique and different from others in many respects.
2. I do my own thing, regardless of what others think.
3. I feel it is important for me to act as an independent person.
4. I act the same way no matter who I am with.
5. My personal identity, independent of others, is very important to me.

***Vaccine acceptance***

Please rate how much you agree or disagree with each of the following statements. (*1=Strongly disagree 7=Strongly agree*)

1. During the pandemic, it has been very important to me to get a vaccine.
2. During the pandemic, it has been very important to me that everyone who can gets vaccinated.
3. I believe vaccines are important in preventing the spread of COVID-19.

***Vaccination status***

Please tell us about your vaccination status:

1. I have not been vaccinated because the COVID-19 vaccine has not been available to me (e.g., there has been a shortage of vaccines in your country, you are not yet eligible to receive one).
2. I have not been vaccinated even though the vaccine has been available to me.
3. I am partially vaccinated.
4. I am fully vaccinated.
5. I am fully vaccinated and have also received a booster shot.
6. __________Other

**Study 2 Measures**

***COVID-19 conspiratorial beliefs***

Same measure as in Study 1.

***Interdependent self-construal***

Same measure as in Study 1.

***Independent*** ***self-construal***

Same measure as in Study 1.

***Prosocial motivation***

Please rate how much you agree or disagree with each of the following statements. (*1=Strongly disagree 7=Strongly agree*)

1. I care about benefiting others.
2. I want to help others.
3. I want to have a positive impact on others.
4. It is important for me to good for others.

***Updated vaccine acceptance***

COVID-19 boosters help restore protection that has waned since previous vaccination by targeting COVID-19 variants that are more transmissible and immune-evading. Please rate how much you agree or disagree with each of the following statements regarding to COVID-19 vaccine booster shots. (*1=Strongly disagree 7=Strongly agree*)

1. It is very important to me to get a COVID-19 booster when I can.
2. It is very important to me that everyone who can get a COVID-19 booster gets one.
3. I believe a COVID-19 booster is important in preventing the spread of COVID-19.

**Study 3 Measures**

***COVID-19 conspiratorial beliefs***

Same measure as in Studies 1 and 2.

***Interdependent self-construal***

Same measure as in Studies 1 and 2.

***Vaccine acceptance***

Same measure as in Study 1.

**Study 4 Measures**

***Self-construal manipulation***

**Interdependent self-construal condition.** Please take a few minutes to write a brief essay about your friendships and camaraderie with other people and how you might foster these relationships. Please write at least 75 words.

**Independent self-construal condition.** Please take a few minutes to write about your unique character and skills, and how you might stand out compared with other people. Please write at least 75 words.

***COVID-19 conspiratorial beliefs***

Same measure as in Studies 1, 2, and 3.

***Vaccine acceptance***

Same measure as in Studies 1 and 3.

**Study 5 Measures**

***Conspiratorial beliefs manipulation. Note: the disease name, dyspeptmeria, is a nod to Cookson et al.’s (2021) work, which examined vaccine attitudes for another fictitious disease, dysomeria.***

Please imagine that you go online and read an article discussing a significant current event. On the next page you will find that article; read it carefully because you will answer questions about it. The page containing the article will not allow you to advance until you have spent at least

one minute reading.

**Low conspiratorial beliefs condition:**

**Dyspeptmeria: An Explainer**

By Staff, CR News

Published 7:41 AM EDT, Tuesday November 16

A novel virus called Dyspeptmeria is spreading rapidly across the world, and there are

official reports explaining the situation.

**What is Dyspeptmeria?**

Dyspeptmeria, an RNA virus, spreads to others when an infected person reaches a certain

viral load, exhaling the virus into the air or on door handles, faucets, and other objects.

Infected individuals have been found on at least three continents, with those infected

suffering from respiratory and digestive issues. The disease initially results in discoloration of the tongue and a mild sore throat but often progresses to fever, diarrhea, and potentially life-threatening intestinal distress.

**Where did Dyspeptmeria come from?**

RNA viruses are found in humans and animals, including livestock, pets, and wildlife such as rats and bats. In general, these viruses rarely cross between species. However,

due to high mutation rates, RNA viruses can leap from animal hosts to humans, as likely

occurred with Dyspeptmeria.

Scientists have agreed that the disease likely originated from contact between bats and

humans at a bat sanctuary in Russia. Some online discussions have found the scientists to

be trustworthy and transparent, noting the robustness of their evidence and conclusions.

Some online discussants have pointed to Russian news articles from eighteen months

ago, reporting an outbreak of a disease in southern Russia, which was also said to involve

tongue and gum discoloration, where the same species of bat is known to be found. The

first infected individuals in the U.S. were discovered in Washington, D.C., where

quarantine procedures failed to prevent its spread. As the virus continues to spread across

the U.S., many believe that media concerns about the dangers of Dyspeptmeria have been

validated, prompting both organizations and individuals to take the necessary

precautions.

**High conspiratorial beliefs condition:**

**Dyspeptmeria: An Explainer**

By Staff, CR News

Published 7:41 AM EDT, Tuesday November 16

A novel virus called Dyspeptmeria is spreading rapidly across the world. Although there

are official reports explaining the situation, there is good reason to question them.

**What is Dyspeptmeria?**

Dyspeptmeria, an RNA virus, spreads to others when an infected person reaches a certain

viral load, exhaling the virus into the air or on door handles, faucets, and other objects.

Infected individuals have been found on at least three continents, with those infected

suffering from respiratory and digestive issues. The disease initially results in

discoloration of the tongue and a mild sore throat but often progresses to fever, diarrhea,

and potentially life-threatening intestinal distress.

**Where did Dyspeptmeria come from?**

RNA viruses are found in humans and animals, including livestock, pets, and wildlife

such as rats and bats. In general, these viruses rarely cross between species. However,

due to high mutation rates, RNA viruses can leap from animal hosts to humans, as likely

occurred with Dyspeptmeria.

While scientists claim that the disease likely originated from contact between bats and

humans at a bat sanctuary in Russia, some online discussions have found the scientists’

evidence and conclusions weak, suggesting that information is being deliberately held

back from the public. Some online discussants instead provide evidence that the Russian

government engineered and released the disease and have pointed to Russian news

articles from eighteen months ago, reporting an outbreak of a disease in southern Russia,

which was also said to involve tongue and gum discoloration. The first infected

individuals in the U.S. were discovered in Washington, D.C., where quarantine

procedures failed to prevent its spread. As the virus continues to spread across the U.S.,

many believe that the media is exaggerating its severity for their own purposes.

***Conspiratorial beliefs manipulation check***

Please rate how much you agree or disagree with each of the following statements. (*1=Strongly disagree 5=Strongly agree*)

1. A lot of information about Dyspeptmeria is deliberately held back from the public.
2. Dyspeptmeria was created and spread by a government organization.
3. The existence of Dyspeptmeria is a hoax perpetuated by the media.

***Self-construal manipulation***

**Interdependent self-construal condition.** Now we're moving on to another part of the study. Please read the next paragraph carefully and highlight all the PRONOUNS found within the paragraph. The pronouns may be singular (e.g., he, she, me, I, you, mine, yours, etc.) or plural (e.g., we, they, our, their, etc.). You can highlight by clicking on a word and then selecting "Highlight".

We go to the city often. Our anticipation fills us as we see the skyscrapers come into view. We allow ourselves to explore every plaza and corner, never letting an attraction escape us. Our voices fill the air and street. We see all the sights, we visit shops, and everywhere we go we see our reflections looking back at us in the glass of a hundred windows. At nightfall we linger, our time in the city almost over. When finally we must leave, we do so knowing that we will

soon return. The city belongs to us.

**Baseline condition.** Now we're moving on to another part of the study. Please read the next paragraph carefully and highlight all the NOUNS found within the paragraph. The nouns may be singular (e.g., anticipation, road, day, etc.) or plural (e.g., reflections, walls, etc.). You can highlight by clicking on a word and then selecting "Highlight".

They go to the city often. His anticipation fills him as he sees the skyscrapers come into view. She allows herself to explore every plaza and corner, never letting an attraction escape her. His voice fills the air and street. She sees all the sights, he visits shops, and everywhere they go they see their reflections looking back at them in the glass of a hundred windows. At nightfall she lingers, their time in the city almost over. When finally they must leave, they do so knowing that they will soon return. The city belongs to them.

***Vaccine acceptance***

Now we would like you to answer questions about the article you read about Dyspeptmeria.

Please imagine that researchers have been working on developing a vaccine to reduce the spread of Dyspeptmeria, and it has just become available. Rate how much you agree or disagree with each of the following statements with regard to the Dyspeptmeria vaccine. (*1=Strongly disagree 7=Strongly agree*)

1. It is very important to me to get a Dyspeptmeria vaccine when I can.
2. It is very important to me that everyone who can a Dyspeptmeria vaccine gets one.
3. I believe Dyspeptmeria vaccine is important in preventing the spread of Dyspeptmeria.

For the essay question, participants were instructed to:

Please take a moment to reflect on the article you read earlier about the Dyspeptmeria and share your thoughts with us. We would like to know how concerned you are about your own health and/or the health of others. You must write at least three sentences describing your thoughts to proceed; otherwise, you cannot advance to the next page.

**Supplementary Analyses**

***Study 1***

**Vaccine acceptance analyses with controls.** We controlled for participants’ age, gender, race, educational level, independent self-construal, and political affiliation (1=Republicans, 2=Democrats, 3=Independent or Other; two dummy variables were created using Republicans as the baseline). We found a significant interactive effect of conspiratorial beliefs and interdependent self-construal on vaccine acceptance (*b* = .31, *SE* = .07, *p <* .001, 95%CI[.18, .44]). When interdependent self-construal was low (-1SD), conspiratorial beliefs were negatively associated with vaccine acceptance (*b* = -1.00, *SE* = .10, *p <* .001, 95%CI[-1.19, -.80]). When interdependent self-construal was high (+1SD), the relationship was weakened (*b* = -.41, *SE* = .09, *p <* .001, 95%CI[-.59, -.23]).

**Vaccination status analyses with controls.** When using vaccination status as the dependent variable, results remained the same after controlling for these variables. Specifically, we found a significant interactive effect of conspiratorial beliefs and interdependent self-construal on the likelihood of being vaccinated (*b* = .45, *SE* = .19, *Z =* 2.35, *p* = .019, 95%CI[.01, .78]). When interdependent self-construal was low (-1SD), conspiratorial beliefs were negatively associated with the likelihood of being vaccinated (*b* = -1.29, *SE* = .29, *Z =* -4.41, *p <* .001, 95%CI[-1.79, -.70]). When interdependent self-construal was high (+1SD), the relationship was weakened (*b* = -.45, *SE* = .21, *Z* = -2.17, *p* = .030, 95%CI[-.85, -.04]).

**Masking acceptance.** There was a significant interactive effect of conspiratorial beliefs and interdependent self-construal on masking acceptance (*b* = .24, *SE* = .06, *p <* .001, 95%CI[.11, .36]). When interdependent self-construal was low (-1SD), conspiratorial beliefs were negatively associated with masking acceptance (*b* = -.82, *SE* = .09, *p <* .001, 95%CI[-1.00, -.65]). When interdependent self-construal was high (+1SD), the relationship was weakened (*b* = -.38, *SE* = .08, *p <* .001, 95%CI[-.54, -.21]; see Figure 9).

***Study 2***

**Updated vaccine acceptance analyses with controls.** We controlled for participants’ age, gender, race, educational level, independent self-construal, political affiliation, and vaccination status. We found a significant interactive effect of conspiratorial beliefs and interdependent self-construal on updated vaccine acceptance (*b* = .14, *SE* = .05, *p* = .006, 95%CI[.04, .24]). When interdependent self-construal was low (-1SD), conspiratorial beliefs were negatively associated with updated vaccine acceptance (*b* = -.73, *SE* = .08, *p* < .001, 95%CI[-.89, -.57]). When interdependent self-construal was high (+1SD), the relationship was weakened (*b* = -.48, *SE* = .08, *p* < .001, 95%CI[-.63, -.33]). We also found significant interactive effect of conspiratorial beliefs and interdependent self-construal on prosocial motivation (*b* = .07, *SE* = .03, *p* = .049, 95%CI[.0002, .14]). When interdependent self-construal was low (-1SD), conspiratorial beliefs were negatively associated with prosocial motivation (*b* = -.26, *SE* = .06, *p* < .001, 95%CI[-.38, -.15]). When interdependent self-construal was high (+1SD), the relationship was weakened (*b* = -.14, *SE* = .05, *p* = .010, 95%CI[-.24, -.03]). Moreover, interdependent self-construal moderated the indirect relationship between conspiratorial beliefs and updated vaccine acceptance through prosocial motivation. When interdependent self-construal was low (-1SD), conspiratorial beliefs were negatively associated with updated vaccine acceptance via prosocial motivation (*IE* = -.06, *SE* = .02, 95%CI[-.10, -.01]). This effect, however, was weakened when interdependent self-construal was high (+1SD; *IE* = -.03, *SE* = .01, 95%CI[-.06, -.01]).

***Study 3***

**Vaccine acceptance analyses with controls.** We controlled for participants’ age, gender, and educational level. We found a significant interactive effect of conspiratorial beliefs and culture on vaccine acceptance (*b* = .76, *SE* = .20, *p* < .001, 95%CI[.37, 1.15]). For participants in the U.K. and U.S., conspiratorial beliefs were negatively associated with vaccine acceptance (*b* = -.79, *SE* = .08, *p* < .001, 95%CI[-.94, -.64]), whereas the effect was not significant (*b* = -.03, *SE* = .19, *p* = .857, 95%CI[-.42, .34]) for participants from India. Moreover, there was a significant interactive effect of conspiratorial beliefs and interdependent self-construal on vaccine acceptance (*b* = .28, *SE* = .06, *p* < .001, 95%CI[.16, .39]). Specifically, the negative effect of conspiratorial beliefs on vaccine acceptance was stronger when interdependent self-construal was low (-1SD; *b* = -1.08, *SE* = .11, *p* < .001, 95%CI[-1.28, -.86]), than when interdependent self-construal was high (+1SD; *b* = -.50, *SE* = .09, *p* < .001, 95%CI[-.69, -.33]). Moreover, the indirect effect differed from zero (*b* = .23, *SE* = .06, 95%CI[.12, .37]), suggesting interdependent self-construal mediated the moderating effect of culture on the relationship between conspiratorial beliefs and vaccine acceptance.

**Masking acceptance.** We found a significant interactive effect of conspiratorial beliefs and culture on masking acceptance (*b* = .50, *SE* = .22, *p* = .024, 95%CI[.06, .93]). For participants in the U.K. and U.S., conspiratorial beliefs were negatively associated with masking acceptance (*b* = -.68, *SE* = .08, *p* < .001, 95%CI[-.83, -.52]), whereas the effect was not significant (*b* = -.18, *SE* = .22, *p* = .425, 95%CI[-.62, .24]; see Figure 10) for participants from India. Moreover, there was a significant interactive effect of conspiratorial beliefs and interdependent self-construal on masking acceptance (*b* = .24, *SE* = .06, *p* < .001, 95%CI[.11, .36]). Specifically, the negative effect of conspiratorial beliefs on masking acceptance was stronger when interdependent self-construal was low (-1SD; *b* = -.93, *SE* = .12, *p* < .001, 95%CI[-1.15, -.69]), than when interdependent self-construal was high (+1SD; *b* = -.42, *SE* = .09, *p* < .001, 95%CI[-.62, -.27]). Moreover, the indirect effect differed from zero (*b* = .21, *SE* = .07, 95%CI[.09, .34]), suggesting interdependent self-construal mediated the moderating effect of culture on the relationship between conspiratorial beliefs and masking acceptance.

**Correlations by country.** We reported the correlations by country in Tables 12a, 12b, and 12c.

***Study 4***

**Vaccine acceptance analyses with controls.** We controlled for participants’ age, gender, race, educational level, political affiliation, and vaccination status. We found an interactive effect of conspiratorial beliefs and the self-construal manipulation on vaccine acceptance (*b* = .19, *SE* = .09, *p* = .029, 95%CI[.02, .36]). In the independent self-construal condition, conspiratorial beliefs were negatively associated with vaccine acceptance (*b* = -.55, *SE* = .07, *p* < .001, 95%CI[-.68, -.41]). In the interdependent self-construal condition, the effect was weakened (*b* = -.36, *SE* = .07, *p* < .001, 95%CI[-.49, -.23]).

**Measures Used in Supplementary Analyses**

***Political identification (Studies 1, 2 and 4)***

In politics, as of today, do you consider yourself a…

1. Republican
2. Democrat
3. Independent
4. __________Other

***Masking acceptance (Studies 1 and 3)***

Please rate how much you agree or disagree with each of the following statements. (*1=Strongly disagree 7=Strongly agree*)

1. During the pandemic, it has been very important to me to wear a mask.
2. During the pandemic, it has been very important to me that everyone wears masks.
3. I believe masks are important in preventing the spread of OVID-.

***Vaccination status (Study 2)***

1. I have not been vaccinated against COVID-19.
2. I am vaccinated against COVID-19 and have NOT received a booster shot.
3. I am vaccinated against COVID-19 and have received one booster shot.
4. I am vaccinated against COVID-19 and have received two booster shots.
5. I am vaccinated against COVID-19 and have received three booster shots.
6. I am vaccinated against COVID-19 and have received four booster shots.
7. I am vaccinated against COVID-19 and have received five booster shots.
8. I am vaccinated against COVID-19 and have received six or more booster shots.

***Vaccination status (Study 4)***

1. I have not been vaccinated even though the vaccine has been available to me.
2. I have received one vaccine shot.
3. I have received two vaccine shots.
4. I have received three vaccine shots.
5. Other __________

**Table 12a**

*Means, Standard Deviations, and Correlations for Variables, Study 3, the U.S.*

|  | Variable | *M* | *SD* | 1 | 2 | 3 | 4 |
| --- | --- | --- | --- | --- | --- | --- | --- |
| 1 | Conspiratorial belief | 2.10 | 1.17 |  |  |  |  |
| 2 | Interdependent self-construal | 4.73 | 1.10 | .03 |  |  |  |
| 3 | Vaccine acceptance | 5.49 | 2.00 | -.53^***^ | .20^*^ |  |  |
| 4 | Age | 39.20 | 10.85 | .08 | -.09 | -.08 |  |
| 5 | Gender | - | - | .06 | .10 | -.06 | .12 |

*Note.* *N* = 156. Gender (0 = Man, 1 = Woman). We excluded one participant who did not identify as a man or a woman for the row of gender.

**Table 12b**

*Means, Standard Deviations, and Correlations for Variables, Study 3, the U.K.*

|  | Variable | *M* | *SD* | 1 | 2 | 3 | 4 |
| --- | --- | --- | --- | --- | --- | --- | --- |
| 1 | Conspiratorial belief | 2.24 | .96 |  |  |  |  |
| 2 | Interdependent self-construal | 4.78 | .90 | .14 |  |  |  |
| 3 | Vaccine acceptance | 5.69 | 1.60 | -.38^***^ | .24^***^ |  |  |
| 4 | Age | 33.92 | 10.10 | .01 | -.08 | -.02 |  |
| 5 | Gender | - | - | .07 | .10 | -.05 | -.09 |

*Note.* *N* = 180. Gender (0 = Man, 1 = Woman).

**Table 12c**

*Means, Standard Deviations, and Correlations for Variables, Study 3, India*

|  | Variable | *M* | *SD* | 1 | 2 | 3 | 4 |
| --- | --- | --- | --- | --- | --- | --- | --- |
| 1 | Conspiratorial belief | 3.90 | .74 |  |  |  |  |
| 2 | Interdependent self-construal | 5.60 | .98 | .72^***^ |  |  |  |
| 3 | Vaccine acceptance | 5.86 | .97 | .51^***^ | .73^***^ |  |  |
| 4 | Age | 29.13 | 4.43 | -.24^**^ | -.01 | .16^*^ |  |
| 5 | Gender | - | - | -.07 | -.02 | -.08 | .08 |

*Note.* *N* = 151. Gender (0 = Man, 1 = Woman).

*Figure 9.* Masking Acceptance as a Function of Conspiratorial Beliefs and Interdependent Self-Construal (Study 1)

*Note:* CB = Conspiratorial beliefs. ISC = Interdependent self-construal. 95% confidence interval error bars were added to the graph.

*Figure 10.* Masking Acceptance as a Function of Conspiratorial Beliefs and Culture (Study 3)

*Note:* CB = Conspiratorial beliefs. 95% confidence interval error bars were added to the graph.
